# Comparison of drug survival on adalimumab, etanercept, golimumab and infliximab in patients with axial spondyloarthritis

**DOI:** 10.1371/journal.pone.0216746

**Published:** 2019-05-30

**Authors:** Monika Hebeisen, Almut Scherer, Raphael Micheroli, Michael J. Nissen, Giorgio Tamborrini, Burkhard Möller, Pascal Zufferey, Pascale Exer, Adrian Ciurea

**Affiliations:** 1 Department of Rheumatology, UniversitätsSpital Zürich, Zurich, Switzerland; 2 Statistics Group, SCQM Foundation, Zurich, Switzerland; 3 Division of Rheumatology, University Hospital Geneva, Geneva, Switzerland; 4 Private Center for Sonography and Rheumatology, Basel, Switzerland; 5 Department of Rheumatology, Immunology and Allergology, Inselspital, Bern, Switzerland; 6 Division of Rheumatology, Centre Hospitalier Universitaire Vaudois, Lausanne, Switzerland; 7 Praxis Rheuma-Basel, Basel, Switzerland; Soroka University Medical Center, ISRAEL

## Abstract

**Objectives:**

To compare drug survival in patients with axial spondyloarthritis treated with different TNF inhibitors in standard dosage.

**Methods:**

Patients fulfilling the Assessment in SpondyloArthritis international Society classification criteria for axial spondyloarthritis in the Swiss Clinical Quality Management cohort were included in this study if a first TNF inhibitor on standard dosage was started after recruitment and if a baseline visit was available. Drug maintenance up to drug discontinuation or dose escalation was compared between TNF inhibitors with multiple adjusted Cox proportional hazards models and multiple imputation for missing baseline covariate data.

**Results:**

A total of 966 patients were included (adalimumab 344, etanercept 237, golimumab 214, infliximab 171). Patients on certolizumab (n = 18) were excluded. Patients starting golimumab had lower disease activity as well as better physical function and quality of life in comparison to patients starting another drug. A higher proportion of patients starting infliximab had a history of extra-articular manifestations. Drug dosage was more often escalated during follow-up in patients treated with infliximab than with subcutaneously administered agents. However, no significant differences in time up to drug discontinuation or dose escalation were observed in multiple adjusted analyses if treatment was initiated after 2009, when all 4 TNF inhibitors were available: hazard ratio for infliximab versus etanercept 1.16 (95% confidence interval 0.80; 1.67), p = 0.44, for golimumab versus etanercept 0.80 (0.58; 1.10), p = 0.17 and for adalimumab versus etanercept 0.93 (0.69; 1.26), p = 0.66.

**Conclusion:**

In axial spondyloarthritis, drug survival with standard doses of different TNF inhibitors is comparable.

## Introduction

Drug survival is a composite measure of effectiveness and safety. It is additionally influenced by the number of alternative treatment options and changes in the population treated over time. Moreover, personal preferences of patients and their physicians, governmental interventions in the health care system and marketing efforts of the pharmaceutical industry may have an impact on drug maintenance. In axial spondyloarthritis (axSpA), several national register studies have demonstrated a better drug retention in patients treated with etanercept (ETA) and adalimumab (ADA) in comparison to infliximab (IFX) [1–3]. In contrast, other studies in axSpA, including our previous analyses, have suggested that the choice of the TNFi did not affect drug survival [4–10]. These results might have been confounded by the fact that discontinuation rates usually increase with later calendar periods, as alternative treatment options arise, as demonstrated for rheumatoid arthritis [11]. Moreover, a differential immunogenicity has been described for the different anti-TNF agents, potentially leading to a gradual loss of effectiveness [12, 13]. We hypothesized that the failure to detect a lower drug retention in patients with IFX in some studies might be due to a higher proportion of patients on IFX presenting with an increase in dosage during follow-up. The aim of this study was to compare drug survival up to dose escalation in axSpA patients treated with different TNFi and to adjust for additional potential confounders not available in previous analyses.

## Materials and methods

### Study population

Patients with a clinical diagnosis of axSpA recruited in the SCQM cohort [14] since 2004 were included in the current study if they fulfilled the Assessment in SpondyloArthritis international Society (ASAS) classification criteria for axSpA [15], if they started a first TNFi approved for this condition after recruitment on a licensed standard dosage and if baseline disease activity information was available. Clinical assessments were performed according to the recommendations of ASAS [16] and visits were scheduled annually after baseline. Intermediate visits were recommended before and 3 months after treatment changes. Scoring of sacroiliac joints allowing for differentiation between nonradiographic axSpA (nr-axSpA) and ankylosing spondylitis (AS) was performed centrally [17]. The study was approved by the Ethics Commission of the Canton of Zurich. Written informed consent was obtained from all patients.

### Drug retention analyses

Medication start and stop dates indicated by the treating rheumatologist were used to estimate the time individual patients maintained their first TNFi treatment. With the introduction of a smartphone application in 2016, SCQM patients can additionally report if the medication information entered by the rheumatologist in the database is correct on a monthly basis. Observations were censored at the last visit or at the last change in TNFi dosage registered in SCQM, whatever occurred last. To account for potential differences in dose escalation between different TNFi (ADA, certolizumab (CER), ETA, golimumab (GOL) and IFX, time to drug discontinuation or dose escalation (referred to as time to dose escalation/stop) was additionally analyzed. Dose escalation of TNFi was defined as either an increase in dose or a shortening of the interval between treatment administrations of >10%.

### Statistical analysis

Baseline characteristics between patients treated with different anti-TNF agents were compared using the Fisher’s exact test for categorical variables and the Mann-Whitney test for continuous variables. Crude time to treatment discontinuation as well as time to dose escalation/stop were described with Kaplan-Meier plots. Log-rank test p-values are provided. Multiple adjusted Cox proportional hazards models were set up to estimate a covariate-adjusted effect of the choice of TNFi on drug maintenance. The following baseline covariates were considered: sex, age, disease duration, calendar period (to account for the number of TNFi at choice at different time-points during follow-up), human leucocyte antigen (HLA) B27, classification status as nr-axSpA vs. AS, co-medication with conventional synthetic anti-rheumatic disease-modifying drugs (csDMARDs), Bath Ankylosing Disease Activity Index (BASDAI), Bath Ankylosing Disease Functional Index (BASFI), elevated C-reactive protein (CRP) status, presence of extra-articular manifestations, current smoking, education (either vocational or university education versus compulsory education), body mass index (BMI ≥30 and >25 versus normal weight, respectively), physical exercise (yes versus no) and interactions between calendar period and TNFi type. To avoid collinearity between selected covariates, adjustment was finally performed for sex, disease duration, classification status, BASDAI, csDMARD co-medication, elevated CRP status, presence of extra-articular manifestations, current smoking, education, calendar period and its interaction with the type of TNFi.

The cox models were fitted using multiple imputation of missing covariate data to account for missing values [18]. Out of 873 treatments, 598 had at least one missing value in one of the 11 variables used. The proportion of missing values per variable varied from 0% to 39% (the latter pertaining to classification status as nr-axSpA versus AS, when a conventional radiograph of the pelvis was not available).

R statistical software (R Development Core Team, 2011) was used for all analyses. All tests were two-sided, with the significance level set to 0.05.

## Results

### Baseline characteristics

A total of 984 patients started a first TNFi with standard dosage after inclusion into the cohort and had a baseline visit. CER was initiated as a first TNFi in only 18 patients and excluded from the analyses. Baseline characteristics of the remaining 966 patients treated with different anti-TNF agents are shown in Table 1 (ADA n = 344, ETA n = 237, GOL n = 214, IFX n = 171). Corresponding to the different time-points of their approval for AS (and later axSpA for some TNFi), the median year of treatment initiation differed between the individual drugs. Patients treated with GOL, a biologic first approved for this indication in Switzerland in 2010, had a lower disease activity as well as a better function, spinal mobility and health-related quality of life at baseline. No significant differences were observed for sex, HLA-B27 positivity, age at disease onset, presence of peripheral arthritis or enthesitis between the individual TNFi. The presence of extra-articular manifestations in patient history affected the choice of the first TNFi: Only 4.2% of patients with inflammatory bowel disease were treated with ETA, while the respective proportion of patients treated with IFX was 18.6% (p<0.001). IFX was most frequently used in combination with csDMARDs (17.0% vs. 13.9% in ADA, 11.8% in ETA and 7.5% in GOL; overall p = 0.03).

**Table 1:** Baseline characteristics at start and reasons for later discontinuation of first TNF inhibitor.

Parameter	N	ADA N = 344	ETA N = 237	GOL N = 214	IFX N = 171	Overall P value
Year TNFi start median, IQR	966	2010 2009;2013	2009 2006;2013	2014 2012;2016	2010 2007; 2012	<0.001
Male sex, %	966	54.1	57.4	54.7	63.2	0.23
AS, %	572	65.9	78.3	62.8	75.2	0.01
Age, years	966	38.9 (11.4)	39.2 (10.5)	38.4 (12.2)	40.2 (11.6)	0.34
Age at first symptoms	966	26.8 (8.3)	26.9 (8.3)	27.2 (8.4)	27.6 (9.1)	0.86
Symptom duration, years	954	12.2 (11.0)	12.3 (9.9)	11.2 (11.4)	12.5 (10.5)	0.11
HLA-B27 pos, %	892	73.9	75.9	79.2	76.1	0.58
BASDAI	795	5.6 (2.0)	5.7 (2.0)	5.3 (2.0)	5.8 (1.8)	0.15
Patient GA	798	6.4 (2.3)	6.7 (2.4)	6.1 (2.3)	6.4 (2.4)	0.08
ASDAS-CRP	746	3.4 (0.9)	3.5 (0.9)	3.2 (0.9)	3.5 (0.9)	0.002
Elevated CRP, %	897	48.1	50.5	45.1	54.2	0.36
BASFI	801	4.0 (2.6)	4.3 (2.4)	3.4 (2.3)	4.5 (2.4)	<0.001
BASMI	817	1.9 (1.9)	2.2 (1.9)	1.7 (1.6)	2.4 (2.0)	0.01
EQ-5D	785	54.4 (21.5)	53.1 (22.6)	60.4 (20.0)	54.0 (21.6)	0.01
Arthritis, %	938	35.4	36.4	34.3	32.9	0.90
Enthesitis, %	937	74.2	73.0	69.5	68.3	0.43
EAM ever, %	802	37.9	38.8	28.0	52.5	<0.001
Uveitis ever, %	865	19.5	20.6	13.9	26.8	0.03
Psoriasis ever, %	771	10.4	13.4	12.2	12.1	0.79
IBD ever, %	857	11.4	4.2	5.8	18.6	<0.001
csDMARD at BL, %	966	13.9	11.8	7.5	17.0	0.03
• methotrexate, %	966	7.8	4.2	3.7	8.2	0.08
• sulfasalazine, %	966	7.3	5.9	3.7	8.2	0.24
• leflunomide, %	966	0.0	1.3	0.5	2.3	0.02
Current smokers, %	765	37.8	31.4	36.8	42.9	0.18

Except where indicated otherwise, values are the mean (standard deviation). ADA = Adalimumab; AS = Ankylosing Spondylitis; ASDAS-CRP = Ankylosing Spondylitis Disease Activity Score using C-reactive protein (CRP) levels; BASDAI = Bath Ankylosing Spondylitis Disease Activity Index; BASFI = Bath Ankylosing Spondylitis Functional Index; BASMI = Bath Ankylosing Spondylitis Metrology Index; BL = baseline; csDMARD = conventional synthetic Disease-Modifying Anti-Rheumatic Drug; EAM = Extra-articular manifestations; EQ-5D = EuroQoL-5domains; ETA = Etanercept; GA = Global assessment; GOL = Golimumab; HLA-B27 = human leucocyte antigen B27; IBD = Inflammatory bowel disease; IFX = Infliximab; TNF = Tumor Necrosis Factor.

### Dose escalation during follow-up

The proportion of patients with dose escalation was analyzed during the observation time in SCQM, excluding patients having experienced both a reduction and an increase in dosage during follow-up (n = 11). It was significantly higher in patients treated with IFX in contrast to TNFi administered subcutaneously (IFX 18% vs. ADA 2%, ETA 0%, GOL 1%; overall p-value <0.001).

### Drug maintenance

Treatment retention analysis was performed in 873 patients, as 93 patients had no database entries after the baseline visit and were therefore censored at treatment start. Baseline characteristics of this large subgroup of patients were comparable to the whole population (Table 2). The TNFi was discontinued in 493 patients during follow-up. The reasons for treatment discontinuation are provided in Table 2.

**Table 2:** Baseline characteristics at start of first TNF inhibitor of patients retained in the drug retention analysis.

Baseline characteristics	N	ADA N = 320	ETA N = 216	GOL N = 183	IFX N = 154	P
Year TNFi start median, IQR	873	2011 (2008; 2013)	2008 (2006; 2012)	2014 2012; 2015)	2009 (2007; 2012)	<0.001
Male sex, %	873	54.1	58.3	56.3	66.9	0.06
AS, %	535	64.7	79.9	64.3	76.0	0.008
Age, years	873	39.2 (11.5)	39.0 (10.4)	38.7 (12.4)	39.8 (11.3)	0.68
Age at first symptoms	864	26.9 (8.3)	26.5 (8.2)	26.7 (8.2)	27.4 (9.0)	0.69
Symptom duration, years	864	12.4 (11.1)	12.4 (9.7)	12.0 (11.7)	12.4 (9.9)	0.49
HLA-B27 pos, %	804	74.0	77.7	79.7	76.1	0.55
BASDAI	726	5.6 (2.0)	5.7 (2.0)	5.2 (2.0)	5.8 (1.8)	0.07
Patient GA	727	6.5 (2.3)	6.7 (2.5)	5.9 (2.3)	6.4 (2.4)	0.01
ASDAS-CRP	683	3.4 (0.9)	3.5 (0.9)	3.1 (0.9)	3.6 (0.9)	<0.001
Elevated CRP, %	815	48.8	50.2	44.6	55.0	0.34
BASFI	730	4.0 (2.7)	4.3 (2.5)	3.3 (2.3)	4.5 (2.4)	<0.001
BASMI	740	2.0 (1.9)	2.2 (1.9)	1.7 (1.7)	2.4 (2.1)	0.01
EQ-5D	714	54.3 (21.8)	52.8 (22.7)	62.0 (19.0)	53.4 (22.2)	<0.001
Arthritis, %	846	35.6	35.2	34.5	30.7	0.76
Enthesitis, %	846	74.8	72.2	69.4	69.6	0.52
EAM ever, %	736	36.9	40.7	28.9	53.9	<0.001
Uveitis ever, %	792	19.4	22.6	12.6	27.7	0.007
Psoriasis ever, %	711	10.4	12.8	12.9	12.8	0.78
IBD ever, %	784	10.8	4.6	6.1	18.2	0.001
csDMARD, %	873	14.4	11.6	6.0	17.5	0.008
• methotrexate, %	873	8.1	4.2	3.8	9.1	0.07
• sulfasalazine, %	873	7.5	6.0	2.2	9.1	0.04
• leflunomide, %	873	0.0	0.9	0.6	1.9	0.04
Current smokers, %	695	38.3	32.0	37.5	42.1	0.32
**Reasons of** **discontinuation**	493					0.01
• Ineffectiveness, %	243	54.1	48.8	47.0	42.1	
• Adverse Events, %	97	17.0	15.7	19.3	30.5	
• Remission, %	50	11.9	5.0	13.2	10.5	
• Other, %	103	17.0	30.6	20.5	16.8	

Except where indicated otherwise, values are the mean (standard deviation). ADA = adalimumab; AS = Ankylosing Spondylitis; ASDAS-CRP = Ankylosing Spondylitis Disease Activity Score using C-reactive protein (CRP) levels; BASDAI = Bath Ankylosing Spondylitis Disease Activity Index; BASFI = Bath Ankylosing Spondylitis Functional Index; BASMI = Bath Ankylosing Spondylitis Metrology Index; csDMARD = conventional synthetic Disease-Modifying Anti-Rheumatic Drug; EAM = Extra-articular manifestations; EQ-5D = EuroQoL-5domains; ETA = etanercept; GA = Global assessment; GOL = golimumab; HLA-B27 = human leucocyte antigen B27; IBD = Inflammatory bowel disease; IFX = infliximab; TNF = Tumor Necrosis Factor.

**Fig 1:**
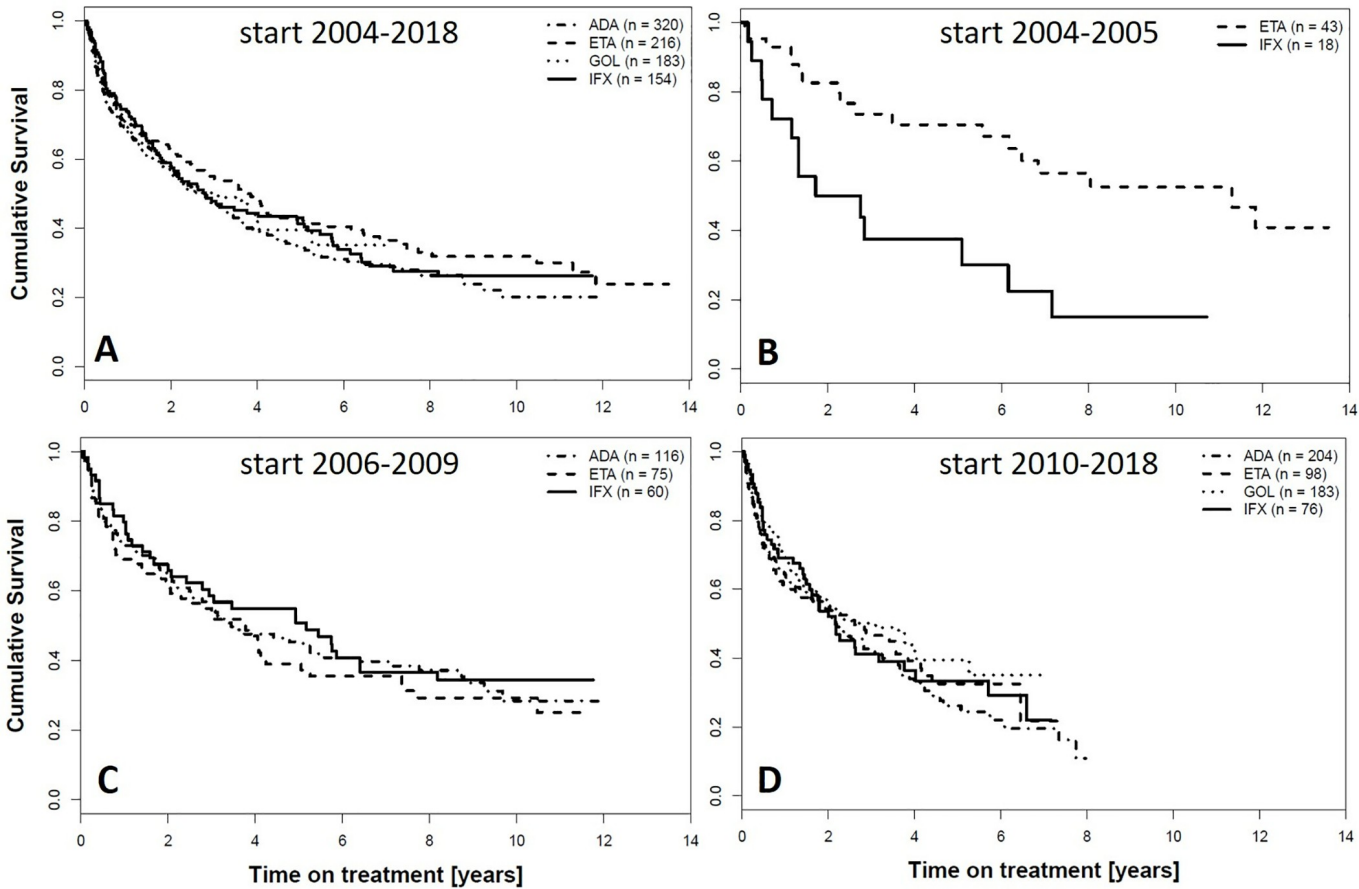
Kaplan-Meier curves showing drug survival of the first TNFi, stratified by the type of TNFi used. A. Treatment initiation during the whole observation time in SCQM. B-D. Stratitication by the number of TNFi available as treatment options at the time-point of treatment initiation. B. ETA and IFX in treatment initiation 2004–2005. C. ADA, ETA and IFX in treatment initiations 2006–2009. D. ADA, ETA, GOL and IFX in treatment initiations after 2009. ADA = adalimumab, ETA = etanercept, GOL = golimumab, IFX = infliximab.

Drug survival is shown for individual TNFi during the whole follow-up in SCQM (2004–2018) in Fig 1A (overall log-rank test p-value 0.23). Binary differences were similarly statistically not significant (ETA-GOL p = 0.45, ETA-IFX p = 0.49, ETA-ADA 0.09, GOL-IFX p = 0.82, GOL-ADA p = 0.59, ADA-IFX p = 0.42). Drug retention is also depicted for individual TNFi after stratification for periods of time corresponding to the number of TNFi available as treatment options (2 TNFi during 2004–2005; 3 TNFi during 2006–2009; at least 4 TNFi 2010–2018, Fig 1B–1D, respectively).

**Fig 2:**
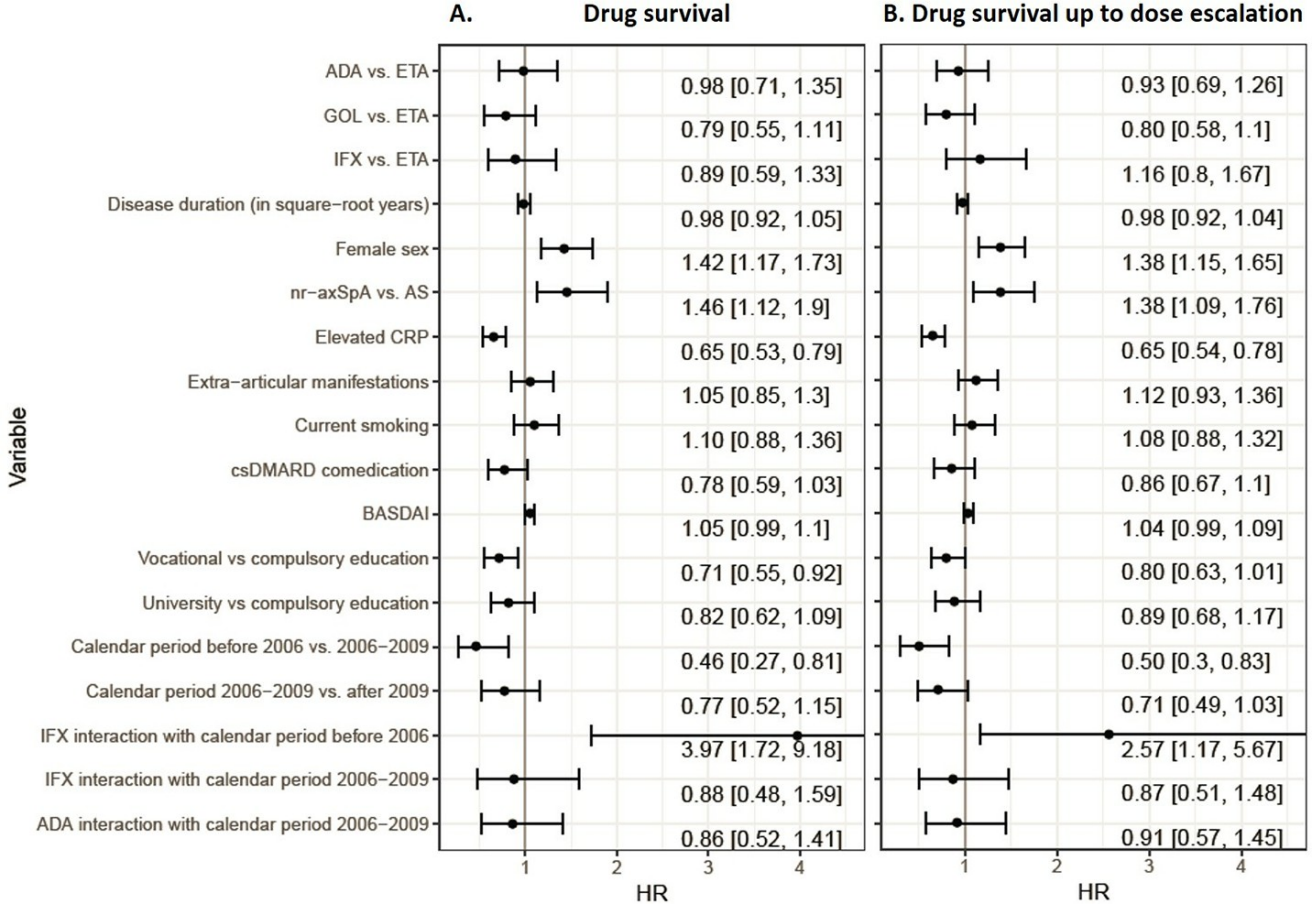
**Multivariable adjusted Cox regression models for drug survival (A) and drug survival up to dose escalation (B) in axSpA patients treated with a first TNFi.** ETA and the period after 2009 are used as references in this models. HRs >1 indicate increased hazard for discontinuation. ADA = adalimumab, AS = ankylosing spondylitis, BASDAI = Bath Ankylosing Spondylitis Disease Activity Index, BASFI = Bath Ankylosing Spondylitis Functional Index, BMI = body mass index, CI = confidence interval, CRP = C-reactive protein, csDMARD = conventional synthetic disease-modifying anti-rheumatic drug, ETA = etanercept, GOL = golimumab, HLA-B27 = Human Leucocyte Antigen B27, HR = hazard ratio, IFX = infliximab, nr-axSpA = nonradiographic axial spondyloarthritis, TNFi = Tumor Necrosis Factor inhibitor.

In multiple adjusted Cox regression analyses, female sex and classification as nr-axSpA were associated with a higher hazard of discontinuation, while elevated CRP levels, vocational versus compulsory education, as well as treatment with ETA in the calendar period before 2006 in contrast to 2006–2009 were associated with a lower hazard of treatment stop (Fig 2A). Comedication with csDMARDs was not found to be associated with a significantly lower hazard of discontinuation (HR 0.78, 95% CI 0.59; 1.03) in this analyses.

Comparable results were found for the complete case analysis (Table 3).

**Table 3:** Multivariable adjusted Cox regression models with complete case data.

	A. Drug survival			B. Drug survival up to dose escalation		
Variable	HR	95% CI	P	HR	95% CI	P
ADA vs. ETA	0.77	0.47; 1.25	0.29	0.77	0.49; 1.23	0.28
GOL vs. ETA	0.57	0.32; 1.02	0.06	0.67	0.39; 1.15	0.15
IFX vs. ETA	0.76	0.41; 1.41	0.39	1.24	0.71; 2.15	0.45
Disease duration (in square-root years)	1.03	0.93; 1.14	0.57	1.02	0.93; 1.11	0.71
Female sex	1.29	0.96; 1.74	0.10	1.18	0.89; 1.56	0.25
nr-axSpA vs. AS	1.30	0.92; 1.84	0.13	1.15	0.83; 1.59	0.40
Elevated CRP	0.84	0.63; 1.13	0.25	0.83	0.64; 1.10	0.19
Extra-articular manifestations	0.94	0.70 ; 1.27	0.71	0.94	0.72 ; 1.24	0.67
Current smoking	0.98	0.73 ; 1.32	0.92	1.04	0.79 ; 1.37	0.76
csDMARD comedication	0.67	0.42 ; 1.06	0.08	0.82	0.55 ; 1.22	0.33
BASDAI	1.04	0.96 ; 1.12	0.37	1.03	0.96 ; 1.10	0.47
Vocational vs compulsory education	0.75	0.51 ; 1.12	0.17	0.83	0.56 ; 1.21	0.33
University vs compulsory education	0.82	0.53 ; 1.29	0.40	0.96	0.62 ; 1.47	0.83
Calendar period before 2006 vs. 2006–2009	0.55	0.26; 1.16	0.12	0.43	0.21; 0.87	0.02
Calendar period 2006–2009 vs. after 2009	0.60	0.33 ; 1.10	0.10	0.65	0.38 ; 1.14	0.13
IFX interaction with calendar period before 2006	2.61	0.70; 9.72	0.15	2.66	0.74; 9.60	0.13
IFX interaction with calendar period 2006–2009	1.15	0.48; 2.74	0.76	0.84	0.38; 1.83	0.66
ADA interaction with calendar period 2006–2009	1.29	0.62; 2.70	0.50	1.18	0.59; 2.35	0.64

Multivariable adjusted Cox regression models with complete case data for treatment retention (A) and time up to dose escalation/stop (B) in axSpA patients treated with a first TNFi. ETA and the period after 2009 are used as references in this model. A total of 347 patients are analyzed. HRs >1 indicate increased hazard for discontinuation. ADA = adalimumab, AS = ankylosing spondylitis, BASDAI = Bath Ankylosing Spondylitis Disease Activity Index, BASFI = Bath Ankylosing Spondylitis Functional Index, BMI = body mass index, CI = confidence interval, CRP = C-reactive protein, csDMARD = conventional synthetic disease-modifying anti-rheumatic drug, ETA = etanercept, GOL = golimumab, HLA-B27 = Human Leucocyte Antigen B27, HR = hazard ratio, IFX = infliximab, nr-axSpA = nonradiographic axial spondyloarthritis, TNFi = Tumor Necrosis Factor inhibitor.

Paralleling the differences in the observed course of individual drug retention after stratification for different time periods in Fig 1B–1D, a significant interaction was found between the calendar period of treatment initiation and the type of TNFi in the adjusted analyses. To allow for an easier interpretation of interactions, the hazard ratios (HR) of treatment discontinuation of different TNFi within a calendar period, as well as the HR of treatment discontinuation of each TNFi in different calendar periods were calculated from main effects and interactions in the model. In contrast to ETA, a trend for a higher discontinuation rate was found for IFX during the period prior to 2006 compared to the following period 2006–2009 (HR 1.84, 95% CI 0.98; 3.46, p = 0.06). A trend for a lower drug retention was detected for all three biologics (ADA, ETA, IFX) for the treatment period after 2009, when compared to the period 2006–2009, although differences reached statistical significance only for ADA. Pairwise comparisons of HR of discontinuation for different monoclonal anti-TNF antibody drugs and ETA by calendar periods are shown in Table 4A. IFX was discontinued more frequently than ETA before 2006, a period when only these two anti-TNF agents were available (hazard ratio (HR) 3.10, 95% confidence interval (CI) 1.52; 6.33, p = 0.002). No significant differences between the discontinuation rates of individual TNFi could be detected at later time-points, with 3 TNFi available after 2006, and >3 after 2009 (Tab.4A).

**Table 4:** Comparison of drug survival as well as drug survival up to dose escalation between different anti-TNF agents, stratified by the period of treatment initiation, in respective multiple adjusted cox regression models.

**Calendar period**	**Before 2006**			**2006–2009**			**After 2009**		
**Available TNFi**	**ETA / IFX**			**ADA / ETA /IFX**			**ADA / ETA / GOL / IFX**		
**A. Drug survival**	**HR**	**95% CI**	**P**	**HR**	**95% CI**	**P**	**HR**	**95% CI**	**P**
IFX vs. ETA	3.10	1.52; 6.33	0.002	0.78	0.50; 1.21	0.27	0.89	0.59; 1.33	0.57
ADA vs. ETA				0.84	0.58; 1.23	0.37	0.98	0.71; 1.35	0.90
GOL vs. ETA							0.79	0.55; 1.11	0.17
**B. Drug survival up to dose escalation**	**HR**	**95% CI**	**P**	**HR**	**95% CI**	**P**	**HR**	**95% CI**	**P**
IFX vs. ETA	2.58	1.30 ; 5.10	0.007	1.00	0.67; 1.49	0.99	1.16	0.80; 1.67	0.44
ADA vs. ETA				0.85	0.59; 1.21	0.37	0.93	0.69; 1.26	0.66
GOL vs. ETA							0.80	0.58; 1.10	0.17

**A.** Summarized data for differences in retention rates between individual TNFi calculated from cox regression model in Fig 2. HRs>1 indicate increased hazard to discontinue first TNFi. **B.** Summarized data for differences in drug maintenance up to dose escalation from model in Table 3. ADA = adalimumab, CI = confidence interval, ETA = etanercept, GOL = golimumab, HR = hazard ratio, IFX = infliximab, TNFi = Tumor Necrosis Factor inhibitor. The analyses were adjusted for comedication with coventional synthetic disease-modifying anti-rheumatic drugs, calendar period of treatment, disease duration, sex, classification status as nonradiographic axial spondyloarthritis versus ankylosing spondylitis, elevated C-reactive protein status, Bath Ankylosing Spondylitis Disease Activity Index, current smoking and education.

### Drug maintenance up to dose escalation

**Fig 3:**
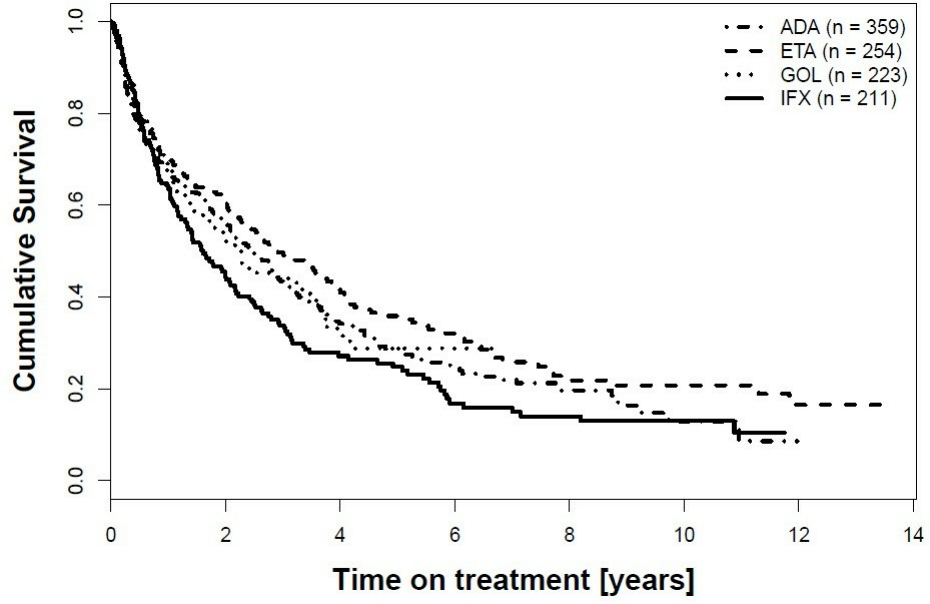
Kaplan-Meier curve showing drug maintenance of the first TNFi up to dose escalation, stratified by the type of TNFi used. ADA = adalimumab, ETA = etanercept, GOL = golimumab, IFX = infliximab.

As these results might have been confounded by the higher rate of dose escalation during follow-up, mainly observed for IFX, we next analyzed drug maintenance up to dose escalation. The results are shown as a Kaplan-Meier plot in Fig 3.

Indeed, a shorter time to dose escalation/stop was found for IFX versus ETA in this unadjusted analysis (log-rank p value 0.01). It was, in contrast, comparable between ADA, ETA and GOL, when compared pairwise. The multiple adjusted analysis confirmed a longer time to dose escalation/stop for IFX versus ETA, but only for the calendar period before 2006 (Table 4B). The model yielded similar retention rates up to dose escalation for all TNFi, when treatment was initiated after 2006 (Table 4B).

## Discussion

Drug retention was compared between ADA, ETA, GOL and IFX in patients with axSpA starting their first TNFi after inclusion into the SCQM axSpA cohort. Retention was comparable when treatment was initiated after 2009, when all four agents were available for treatment. A significant difference in discontinuation rate was only found in patients who started treatment before 2006, when only ETA and IFX were approved for this indication in Switzerland, although this observation is based on a rather limited number of patients, as the cohort had been initiated late in 2004. We have only considered IFX treatments with the registered standard dose of 5 mg/kg every 8 weeks, eliminating the possibility that the shorter drug survival on IFX during this calendar period might be due to the usage of the lower dose registered for rheumatoid arthritis. While in psoriatic arthritis a lower starting dose did not affect drug survival or response in Icelandic and Danish patients [19], this issue has not been sufficiently investigated in axSpA to date. Whether a differential potential of immunogenicity [20] might explain the differences in drug survival between ETA and IFX before 2006 remains unclear, as serum samples to assess the presence of anti-drug antibodies were not available and a causal association would still be difficult to demonstrate [21]. The observation that a higher proportion of patients treated with IFX were co-treated with a csDMARD, might have levelled potential differences in the longer term [2, 8]. The finding that comedication with csDMARDs did not significantly affect drug retention of TNFi is in line with current international axSpA treatment recommendations [22]. Our study confirms the importance of adjusting drug survival analyses for the year or period of treatment initiation [11]. Drug survival on ETA and on ADA decreased over time, paralleling the increasing number of available anti-TNF treatment options, corroborating findings in rheumatoid arthritis [11]. In contrast, a longer drug maintenance on IFX was observed for treatments initiated in the period 2006–2009, compared to treatments started before 2006. The reason for this finding remains unknown, but might involve channeling of particular patients to treatment with IFX and accordingly more regular clinical visits, in contrast to treatment with subcutaneously self-administered anti-TNF agents. Channeling might have also involved patients with extra-articular manifestations, as mirrored in the differences observed in the baseline characteristics. The antagonistic course of drug maintenance over time for IFX versus other anti-TNF agents contributed to the alignment of drug retention rates in later time periods. Drug retention rates were also comparable between the TNFi inhibitors during these periods after accounting for differences in the proportion of patients with dose escalation in the multiple adjusted analysis. The unadjusted analysis had, in contrast, revealed a significantly lower time to drug escalation/stop for IFX versus ETA over the whole follow-up in SCQM.

The main predictors of enhanced drug survival in our analysis were male versus female sex, classification as AS versus nr-axSpA, elevated baseline CRP levels as well as vocational versus compulsory education. We acknowledge the fact that residual confounding in the context of this observational study may still exist. Our analysis points to changes in the axSpA population starting a first TNFi over time, as patients initiating GOL, licensed in 2010, had a significantly lower disease activity and a better spinal mobility and function. Only a minority of patients initiated CER since its registration for axSpA in 2013, indicating that it is mainly used as a second-line TNFi in Switzerland. This precluded the inclusion of CER in the comparison of drug survival of different TNFi.

## Conclusion

Time to treatment discontinuation and time to dose escalation/stop were both comparable for ADA, ETA, GOL and IFX in adjusted analyses in the period of time when all four anti-TNF agents were available on the market.
